# P-1975. Detection of SARS-CoV-2 Specific Secretory IgA and Neutralizing Antibodies in Nasal Secretions of Seronegative Individuals Previously Exposed to COVID-19

**DOI:** 10.1093/ofid/ofae631.2133

**Published:** 2025-01-29

**Authors:** Jason Shin Chwa, Minjun Kim, Yesun Lee, Wesley A Cheng, Yunho Shin, Jaycee Jumarang, Jeffrey Bender, Pia S Pannaraj

**Affiliations:** Keck School of Medicine, University of Southern California, Los Angeles, CA, United States of America, Arcadia, California; Division of Infectious Diseases, Department of Pediatrics, University of California San Diego, San Diego, CA, United States of America, San Diego, California; University of California San Diego, San Diego, California; University of California San Diego, San Diego, California; Children’s Hospital Los Angeles, Los Angeles, California; University of California San Diego, San Diego, California; Children's Hospital Los Angeles, Los Angeles, California; University of California San Diego, San Diego, California

## Abstract

**Background:**

Mucosal immunity may contribute to clearing SARS-CoV-2 infection prior to systemic infection. We describe detection of SARS-CoV-2 specific nasal mucosal antibodies in a group of exposed household individuals that evaded systemic infection and remained seronegative.

**Figure 1.**
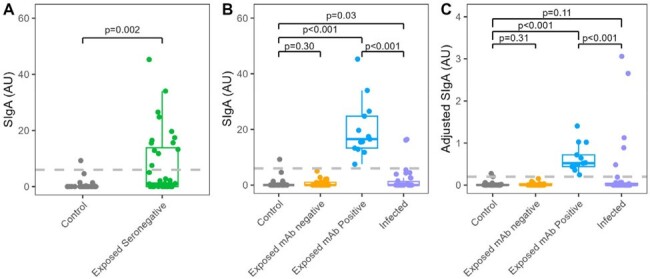
SARS-CoV-2-specific nasal SIgA.

SARS-CoV-2-specific nasal SIgA in control and exposed participants (A). Positive thresholds of 0.20 and 6.30 AU/mL were calculated for unadjusted and adjusted SIgA, respectively, based on mean + 3 standard deviation of the control samples. Exposed participants were stratified by the positive threshold and unadjusted (B) SIgA and adjusted (C) SIgA were compared to controls and infected participants. Wilcoxon rank-sum tests determined if medians differed significantly.

**Methods:**

Between June 2020 and February 2023, nasopharyngeal swabs (NPS) samples and acute and convalescent blood were collected from individuals exposed to a SARS-CoV-2-confirmed household member. Nasal secretory IgA (SIgA) antibodies targeting the SARS-CoV-2 spike protein were measured using a modified ELISA.

**Figure 2.**
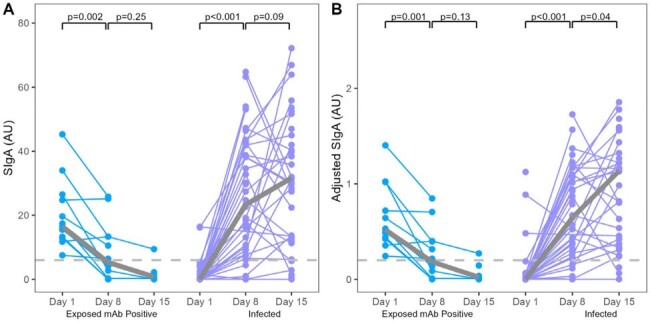
SARS-CoV-2-specific nasal unadjusted (A) and adjusted (B) SIgA levels at Day 1 (enrollment), Day 8, and Day 15 for exposed seronegative with positive nasal SIgA and infected participants.

SARS-CoV-2-specific nasal unadjusted (A) and adjusted (B) SIgA levels at Day 1 (enrollment), Day 8, and Day 15 for exposed seronegative with positive nasal SIgA and infected participants. Wilcoxon signed-rank tests were used to determine if medians (gray) differed significantly.

**Results:**

Of 36 exposed individuals without SARS-CoV-2 detected by RT-PCR of NPS specimens and seronegative for SARS-CoV-2-specific IgG at acute and convalescent visits, 13 (36.1%) had positive SARS-CoV-2-specific SIgA levels detected in the nasal mucosa. These individuals had significantly higher nasal SIgA (median 0.52 AU/mL) compared with never-exposed, never-infected controls (n=29, 0.001 AU/mL) and infected-family members (n=43, 0.0002 AU/mL) during the acute visit, respectively (both P< 0.001, Figure 1). The nasal SARS-CoV-2-specific SIgA decreased rapidly over two weeks in the exposed seronegative individuals (Day 1 [0.52 AU/mL] vs. Day 8 [0.18 AU/mL] vs. Day 15 [median 0.02 AU/mL], P = 0.001 and 0.13, respectively, Figure 2). In contrast, SIgA increased in infected family members (Day 1 [0.0002 AU/mL] vs. Day 8 [0.65 AU/mL] vs. Day 15 [median 1.14 AU/mL], P < 0.001 and P = 0.09, respectively, Figure 2).

**Conclusion:**

Transient nasal SARS-CoV-2-specific SIgA detected in exposed, seronegative individuals may have a protective role in preventing systemic infection. Elucidating factors associated with the induction of nasal SIgA response that prevent systemic invasion may inform infection and transmission prevention strategies.

**Disclosures:**

Pia S. Pannaraj, MD, MPH, AstraZeneca: Grant/Research Support|Pfizer: Grant/Research Support

